# Current status in spatiotemporal analysis of contrast‐based perfusion MRI

**DOI:** 10.1002/mrm.29906

**Published:** 2023-11-06

**Authors:** Eve S. Shalom, Amirul Khan, Sven Van Loo, Steven P. Sourbron

**Affiliations:** ^1^ School of Physics and Astronomy University of Leeds Leeds UK; ^2^ Division of Clinical Medicine University of Sheffield Sheffield UK; ^3^ School of Civil Engineering University of Leeds Leeds UK; ^4^ Department of Applied Physics Ghent University Ghent Belgium

**Keywords:** DCE‐MRI, DSC‐MRI, perfusion, spatiotemporal modeling, tracer kinetics

## Abstract

In perfusion MRI, image voxels form a spatially organized network of systems, all exchanging indicator with their immediate neighbors. Yet the current paradigm for perfusion MRI analysis treats all voxels or regions‐of‐interest as isolated systems supplied by a single global source. This simplification not only leads to long‐recognized systematic errors but also fails to leverage the embedded spatial structure within the data. Since the early 2000s, a variety of models and implementations have been proposed to analyze systems with between‐voxel interactions. In general, this leads to large and connected numerical inverse problems that are intractible with conventional computational methods. With recent advances in machine learning, however, these approaches are becoming practically feasible, opening up the way for a paradigm shift in the approach to perfusion MRI. This paper seeks to review the work in spatiotemporal modelling of perfusion MRI using a coherent, harmonized nomenclature and notation, with clear physical definitions and assumptions. The aim is to introduce clarity in the state‐of‐the‐art of this promising new approach to perfusion MRI, and help to identify gaps of knowledge and priorities for future research.

## 1 INTRODUCTION

Perfusion MRI includes the subfields $T_{1}$‐weighted dynamic contrast‐enhanced MRI (DCE‐MRI),^1^, ^2^ $T_{2}^{*}$‐weighted dynamic susceptibility contrast MRI (DSC‐MRI),^3^, ^4^ and arterial spin labeling (ASL).^5^, ^6^, ^7^ All three methods use an indicator which modifies the MRI signal in proportion to its concentration—either MR contrast agents (DCE‐MRI or DSC‐MRI) or magnetically labeled water (ASL). Rapid dynamic MRI is then used to track the spatiotemporal variations in signal induced by the indicator. After deriving indicator concentration from the measured signal changes, these methods then apply pharmacokinetic (PK) models to obtain maps or region‐of‐interest‐ (ROI) based measurements of perfusion parameters. This review discusses advanced PK modeling and therefore applies to DCE‐MRI, DSC‐MRI, and ASL alike.

The conventional approach to PK modeling in perfusion MRI describes the concentration in each voxel or ROI independently by a one‐dimensional (1D) (temporal) PK model. Conceptually this builds on the fundamental assumption that each voxel or ROI acts as an isolated system with a single, global inlet of tracer.^2^, ^8^, ^9^, ^10^, ^11^ The concentration in the inlet is typically assumed to be known and referred to as the arterial input function (AIF). This assumption effectively separates the problem of modeling a single large four‐dimensional (4D) dataset into a large number of small and independent 1D problems. This makes the analysis highly scalable, parallelizable, and computationally efficient. On the other hand, the assumption is obviously invalid and it has been known for over 20 years that this leads to significant systematic errors.^2^, ^12^, ^13^, ^14^, ^15^, ^16^

In principle, the problem can be resolved by dropping the isolated‐systems assumption and modeling all voxels in the imaged volume as connected systems that all exchange directly with their neighbors.^17^, ^18^, ^19^ Unfortunately, this approach presents significant computational challenges that have so far proven insurmountable. Yet with the increase in computational power and the advance of machine‐learning,^20^, ^21^ solutions are becoming practically feasible. New approaches to spatiotemporal modeling of DCE‐MRI are increasingly proposed, but comparing methods and models between papers presents a significant challenge due to differences in physical concepts, terminology and notations.

The aim of this review is to summarize all relevant developments on spatiotemporal PK modeling of perfusion MRI data in a common framework. This will establish a firm foundation for future developments, facilitate identification of knowledge gaps, and lower the barrier for entry in the field for new researchers.

## 2 HISTORY AND SCOPE

The discussion about the foundations of perfusion MRI is as old as the field itself. In 1990, Henkelman^17^ argued that the very definition of perfusion as inflow per unit volume is not physically justifiable in an imaging setting because fluid flow scales with area rather than volume. While undeniably true, these objections were largely ignored until the early 2000's when Thacker et al.^18^ presented a technique for DCE‐MRI that introduced the concept of cerebral blood flow orientation in terms of spatial gradients in mean transit time. Similar ideas were proposed by Christensen et al.^22^ in DSC‐MRI using the gradient of arterial delay times to derive information about the directionality and orientation of perfusion. Within ASL, an experimental approach for measuring perfusion orientation was proposed which involved labeling planes in different orientations.^23^ From a different angle, the idea of spatial coherence between neighboring voxels has been exploited to estimate AIFs from tissue‐level data by a joint fitting of multiple voxels.^24^, ^25^, ^26^

While these ideas go some way to demonstrate the potential of using spatial information in the analysis, they are limited by the lack of a clear underlying theoretical framework that can be used to build models of spatiotemporal indicator propagation. Since the early 2010's increasing numbers of papers have proposed concepts borrowed from continuum mechanics, computational fluid dynamics or porous media theory to build spatiotemporal generalizations of classic 1D PK models. The earliest proposal dates back to Pellerin et al.^27^ using a spatial model of intervoxel diffusion but retaining the concept of a global AIF to model indicator delivery to the voxel through the vasculature. A first step toward a more general formulation, albeit conceptually confused, can be found in a self‐published report from 2013.^28^

In 2014 these ideas were placed on a more rigorous footing including also multi‐compartmental systems.^19^ One of the consequences is the natural emergence of a formal definition of perfusion (F, in units of mL/min/mL) as the divergence of the arterial flow ($f^{\;a}$, in units of mL/min/cm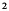):

(1) $$F=-\nabla \cdot f^{\;a}.$$

In words, this states that the perfusion of a piece of tissue is the part of the arterial flow into the tissue that is converted into venous flow out of the tissue. In particular, this does not include the contribution of blood vessels (arterial or venous) that pass through the tissue without feeding its capillaries. This formal definition therefore correctly formalizes the true physiological notion of perfusion as “feeding flow” or “capillary flow” into a given tissue. Moreover, since divergences scale with volume, this fully resolves Henkelman's original objection^17^ to the conventional definition of tissue perfusion as inflow per unit volume.

The main barrier to a more widespread adoption of spatiotemporal models is the computational challenges in applying these to the *inverse* problem of deriving perfusion parameters from data. While the number of free parameters *per voxel* is similar to standard 1D models, the voxels can no longer be solved independently. Spatiotemporal models therefore present a single global inverse problem with, for a typical three‐dimensional (3D) time series, millions of free parameters. And while the problem is linear for the simplest one‐compartmental spatiotemporal models, it is nonlinear in the more general setting of multi‐compartment models.

The scope of this review is therefore restricted to studies applying spatiotemporal modeling to the inverse problem. This excludes a substantial body of the literature using spatiotemporal models as forward models to simulate data. Often these papers aim to generate digital reference objects to investigate and quantify the error caused by neglecting spatial coherence. An example is a study by Barnes et al.^29^ investigating the impact of intra‐voxel diffusion on the accuracy of conventional DCE‐MRI parameters. Another example is a model of the circulation designed to determine the accuracy of conventional perfusion analysis as a function of ROI size.^16^ Incidentally, the latter provides in silico support for Henkelman's objection, showing that the classic definition of perfusion creates a dependence on voxel size, with increasing bias for smaller voxels. Another application of computational fluid dynamics‐type forward models is to help predict and understand drug delivery to tissues, informed by DCE‐MRI data.^30^, ^31^, ^32^ Often these models are multiscale, coupling flow in large blood vessels to microvascular flow and interstitial transport. This is part of a wider literature on multiscale computational modeling of the circulation and biological transport mechanisms.^33^ While these models may be informed by perfusion MRI, they are out of scope for this review unless the models are used to fit the spatial perfusion parameter fields from measured data.

## 3 MODEL CLASSIFICATION

**FIGURE 1:**
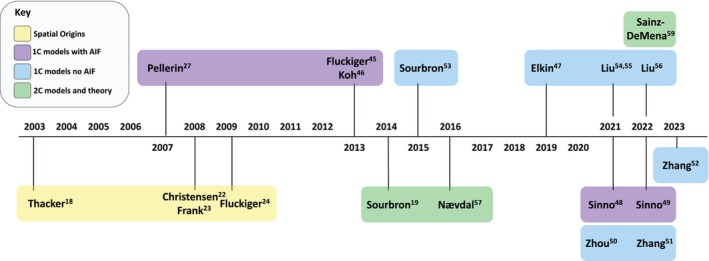
Timeline of contributions within the literature landscape leading toward developing spatiotemporal tracer kinetics. The studies listed are grouped by the theme of the work or model applied using distinct colors as indicated by the key.

**FIGURE 2:**
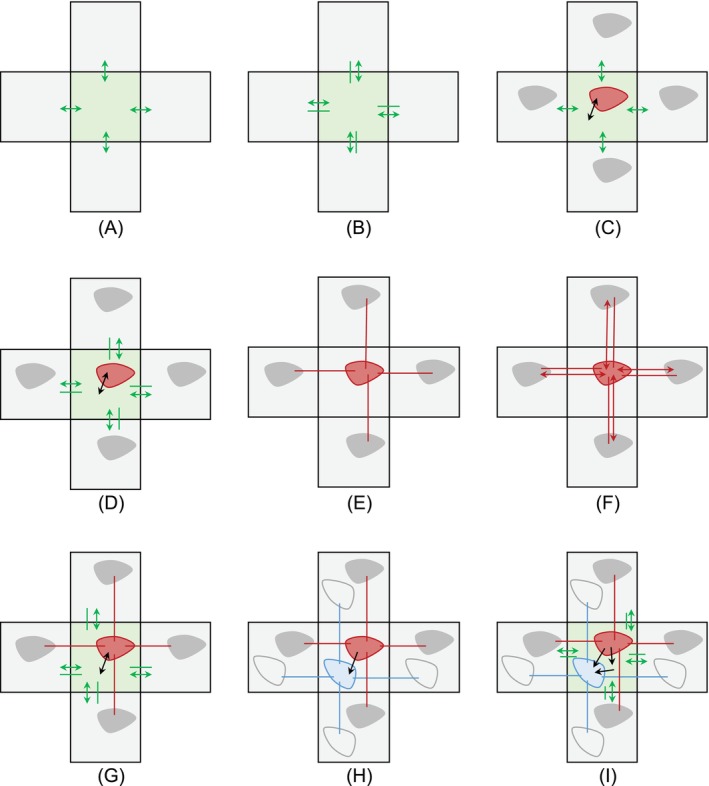
Diagrams of the nine spatiotemporal models proposed in the literature (model equations are given in Table 1). Each model is illustrated for a central voxel and four neighbors, with interstitial (green) and/or vascular compartments (red for arterial or total blood compartments, and blue for venous). Solid colored lines and double‐ended arrows show between‐voxel transport by convection and diffusion, respectively, within a given compartment. Black arrows show within‐voxel exchange between different compartments. Shown are (A) A one‐compartment system with interstitial diffusion; (B) A one‐compartment system with interstitial convection and diffusion; (C) A one‐compartment system with interstitial diffusion and a vascular input; (D) A one‐compartment system with interstitial convection and diffusion and a vascular input; (E) A one‐compartment system with vascular convection; (F) A one‐compartment system with vascular convection and diffusion; (G) A two‐compartment system with vascular convection, interstitial convection and diffusion with bidirectional exchange; (H) A two‐compartment system with vascular convection and mono‐directional exchange; (I) A three‐compartment system with interstitial convection and diffusion, vascular convection, and directional exchange.

A timeline of the publications in scope for this review is shown in Figure 1. Comparison of the model architectures described in these papers reveals nine nested spatiotemporal models of increasing complexity, illustrated in Figure 2 and defined using harmonized notations in Table 1. These nine nested models can be classified as either one‐, two‐ or three‐compartment models depending on the number of distinct compartments in each tissue voxel. Within each group they can be further differentiated based on (1) the transport mechanisms described, such as diffusion, convection, or exchange; (2) the compartment types such as interstitial, arterial, or venous space; and (3) whether an external input function is utilized.

**TABLE 1:** Summary of general system types for spatiotemporal tracer kinetics.

Model	Figure	Dimension	Studies	Transport equations
1C interstitial diffusion	2A	3D	Koh et al.^46^	$\frac{\partial C^{e}}{\partial t}=D^{e}\nabla^{2}C^{e}$
1C interstitial convection/ diffusion	2B	3D	Elkin et al.^47^	$\frac{\partial C^{e}}{\partial t}=D^{e}\nabla^{2}C^{e}-\nabla \cdot u^{e}C^{e}$
1C interstitial diffusion with vascular input	2C	2D	Pellerin et al.^27^ and Fluckiger et al.^45^	$\frac{\partial C^{e}}{\partial t}=K^{\text{trans}}\left(c_{in}-\frac{C^{e}}{v_{e}}\right)+\nabla \cdot D^{e}\nabla C^{e}$
1C interstitial convection/ diffusion with vascular input	2D	2D	Sinno et al.^48^, ^49^	$\frac{\partial C^{e}}{\partial t}=K^{\text{trans}}\left(c_{in}-\frac{C^{e}}{v_{e}}\right)+\nabla \cdot D^{e}\nabla C^{e}-\nabla \cdot u^{e}C^{e}$
1C vascular convection	2E	3D	Zhou et al.^50^ and Zhang et al.^51^, ^52^	$\frac{\partial C^{p}}{\partial t}=-\nabla \cdot u^{p}C^{p}$
1C vascular convection/ diffusion	2F	2D 3D	Zhang et al.,^51^ Sourbron^53^ and Liu et al.^54^, ^55^, ^56^	$\frac{\partial C^{p}}{\partial t}=-\nabla \cdot u^{p}C^{p}+\nabla \cdot D^{p}\nabla C^{p}$
2C interstitial convection/ diffusion with vascular convection and exchange	2G	N/A	Sourbron^19^	$\frac{\partial C^{p}}{\partial t}=-\nabla \cdot u^{p}C^{p}-K^{ep}C^{p}+K^{pe}C^{e}$ $\frac{\partial C^{e}}{\partial t}=-\nabla \cdot u^{e}C^{e}+\nabla \cdot D^{e}\nabla C^{e}+K^{ep}C^{p}-K^{pe}C^{e}$
2C vascular convection and exchange	2H	2D	Nævdal et al.^57^ 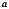	$\frac{\partial C^{a}}{\partial t}=-\nabla \cdot u^{a}C^{a}-K^{va}C^{a}$ $\frac{\partial C^{v}}{\partial t}=-\nabla \cdot u^{v}C^{v}+K^{va}C^{a}$
3C interstitial convection/ diffusion with vascular convection and exchange	2I	N/A	Sourbron^19^	$\frac{\partial C^{a}}{\partial t}=-\nabla \cdot u^{a}C^{a}-K^{va}C^{a}-K^{ea}C^{a}$ $\frac{\partial C^{v}}{\partial t}=-\nabla \cdot u^{v}C^{v}+K^{va}C^{a}+K^{ve}C^{e}$ $\frac{\partial C^{e}}{\partial t}=-\nabla \cdot u^{e}C^{e}+\nabla \cdot D^{e}\nabla C^{e}+K^{ea}C^{a}-K^{ve}C^{e}$

*Notes*: Models are named from the transport mechanisms and input type. All models are defined diagrammatically in Figure 2, with each specific sub‐figure indicated here. The transport equations are shown for each model. Studies from the literature concerning the theory or implementation of specific model types are detailed. Cases that utilize different but equivalent equations from the presented models are tagged with superscript letter “a.” Where implementations are available the dimension of the method is shown, any models tagged N/A are purely theoretical.

The symbols and notations in this paper have been modified from the original publications and harmonized as shown in Table 1 following the definitions in Reference 19. The aim is to reveal the structural differences and similarities between models more clearly. The total tissue concentration, $C(\vec{r},t)$, is a directly measurable quantity and is defined as number of contrast agent molecules per volume of tissue (mmol/mL). If the tissue is built up of multiple compartments, the contribution of a compartment γ to the tissue concentration is given as $C^{\gamma }(\vec{r},t)$, and physically defined as the number of contrast agent molecules in the compartment γ, relative to the volume of the entire tissue (mmol/mL). Examples are the tissue concentration in interstitium $C^{e}(\vec{r},t)$, plasma $C^{p}(\vec{r},t)$, arteries $C^{a}(\vec{r},t)$ or veins $C^{v}(\vec{r},t)$. With this definition, the total tissue concentration $C(\vec{r},t)$ is always the direct sum of the concentrations in the individual compartments. For instance, if a tissue is modelled as consisting of plasma and interstitial compartments, the total tissue concentration is:

(2) $$C(\vec{r},t)=C^{p}(\vec{r},t)+C^{e}(\vec{r},t).$$

The volume fractions do not appear in these equations because concentrations are defined relative to the total tissue volume rather than the compartmental volume. While the equations can be recast to an alternative picture involving volume fractions, flows, and perfusion explicitly (see Reference 19 for details), this introduces additional free parameters that then have to be constrained by adding new constraints. The total blood flow per unit surface area (f, in units of mL/min/cm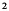) is defined from velocity (u, in units of cm/min) and volume fraction (v, in units of mL/mL), by f=vu. As f is incompressible the systems are constrained as:

(3) ∇·f=v∇·u+u·∇v=0.

Using tissue concentrations up front simplifies the equations and numerical challenges, and ensures the models are defined using the least number of free parameters. After solving for the models in this picture, any missing markers such as volume fraction, perfusion or blood flow can then be derived as described in Reference 19.

The indicator in a compartment γ is transported *between* voxels by velocity fields, $u^{\gamma }(\vec{r})$, and diffusion fields, $D^{\gamma }(\vec{r})$. Indicator exchange within a voxel between compartments β and γ is denoted by rate constants $K^{\gamma \beta }(\vec{r})$, describing exchange from β to γ. Some models have an AIF, $c_{in}(t)$, without a positional coordinate $\vec{r}$, that represents a global vascular input.

The following sections provide a more detailed description of the nine models identified including numerical implementations, and important results. For clarity, the models are detailed in order of increasing model complexity.

## 4 ONE‐COMPARTMENT MODELS

In one‐compartment systems, the compartmental tracer concentration is simply the tissue concentration and is therefore directly accessible through measurement. The implications are that one‐compartment models with convection and diffusion can be recast as a first‐order linear system of equations. The majority of the work on inverse approaches for spatiotemporal models has focused on one‐compartment systems. These effectively describe the voxel as a single compartment with a uniform concentration, and model the exchange of indicator between voxels using diffusion and convection either separately or concurrently. Beyond the precise type of contrast mechanisms, these models differ in the physical compartment that is modeled (intravascular or extravascular), or, equivalently, which tissue spaces are assumed to carry negligible amounts of indicator.

An increasing body of evidence using forward models has demonstrated that ignoring between‐voxel interstitial exchange can lead to significant bias on parameters such as $K^{\text{trans}}$.^29^, ^34^, ^35^, ^36^, ^37^, ^38^ Initial developments in spatiotemporal analysis of perfusion MRI therefore aimed to eliminate this bias by modeling interstitial convection and diffusion. Additionally, the introduction of interstitial convection enables the accurate representation of tumor regions with significant interstitial fluid pressure gradients that drive detectable advective transport.^39^, ^40^, ^41^, ^42^ Only more recently, attention has turned to spatiotemporal models of vascular transport by convection and diffusion.

### 4.1 Interstitial diffusion

Pellerin et al.^27^ introduced a one‐compartment model with interstitial diffusion and a global vascular input function (Figure 2C). The model introduces a new interstitial diffusion parameter, $D^{e}$, which acts to transport contrast agent through the interstitium between adjacent voxels. The model does not incorporate vascular transport between voxels, instead retaining the assumption that contrast agent is delivered to the voxel through a global AIF. Effectively the model, therefore, extends the standard Tofts model^8^, ^9^ with between‐voxel diffusion in the interstitial space. As in the standard Tofts model it is assumed that the concentration in the plasma space is negligible compared to that in the interstitial space, so the measured concentration is made up of the interstitial concentration only ($C=C^{e}$).

Pellerin et al.^27^ reduce the computational challenge of the inverse problem by considering a two‐dimensional (2D) system only, and assuming $D^{e}$ everywhere is a known parameter. All $D^{e}$ values were fixed to a constant (in the simulations) or spatially dependent but derived from a measured apparent diffusion coefficient of water (in data). Optimization was implemented using a simulated annealing algorithm,^43^ a stochastic optimization method that improves parameter recovery in systems with local minima.^44^

Experiments include a synthetic dataset and 2D slices of mouse DCE‐MRI data. The synthetic data modeled 2D circular tumor with a highly perfused rim and necrotic core. $K^{\text{trans}}$ was defined to be zero at the core so the only means of contrast agent transport in the core is via interstitial diffusion. The conventional Tofts model produced unphysical $v_{e}>1$ in the necrotic core, underestimated $K^{\text{trans}}$ in the rim, and overestimated it in the core. These biases disappear after adding the interstitial diffusion terms, allowing a nonvascular transport pathway to the tumor core.

In 2013,^45^ the same team reduced the computational complexity of the model by assuming the differences in $v^{e}$ and the diffusion coefficient between adjacent voxels are negligible. These assumptions decouple the equations of individual voxels, allowing for a voxel‐by‐voxel analysis of the data. This also implies that the interstitial diffusivity of the contrast agent can be fitted as a free parameter.

The result is a drastic reduction in computation time compared to the original model from Pellerin et al.^27^ on the same reference object and using the same optimization method, computation time was reduced from 70 h to 52 s—almost reaching the efficiency of standard Tofts modeling (11 s). Unfortunately, the results also showed large spatial gradients in diffusivity and $K^{\text{trans}}$, indicating that the assumptions do not capture the true behavior of the system. Additionally, due to the voxel‐wise fitting approach the control over global contrast agent conservation is eliminated. This drawback is recognized within the work,^45^ and it is proposed that future iterations of the method should seek to enforce global mass conservation.

Also in 2013, Koh et al.^46^ proposed a one‐compartment model with interstitial diffusion (Figure 2A). This simplifies the model proposed by Pellerin et al.,^27^ by removing contrast agent delivery through a global input followed by extravasation. As such, between‐voxel diffusion remains the sole mechanism for indicator transport through the system. The model equations can in principle be solved directly for the diffusion coefficient by dividing the time‐derivative of the concentration by its Laplacian. Experiments for this method^46^ included 14 sets of 3D mice xenograft DCE‐MRI data of varying cancer types. In practice, stability in the presence of noisy data was improved by clustering voxels with similar contrast‐enhancement patterns, and solving for a single diffusion coefficient in each cluster. While this approach is obviously limited by the strong assumption of diffusion‐only transport, it presents an elegant solution for areas such as homogeneous necrotic tumor cores where these assumptions are justified.

### 4.2 Interstitial convection and diffusion

In 2019, Elkin et al.^47^ introduced a one‐compartment model with interstitial convection and diffusion (Figure 2B). The model applies interstitial diffusion and convection parameters to distribute contrast agent through the interstitium between adjoining voxels. A global vascular input is not included in the model, effectively assuming that all transport between voxels takes place via the interstitium.

Elkin et al.^47^ reduce the scale of the inverse problem by asserting the contrast agent mass density can be written as a function of velocity. For optimization, an operator splitting method followed by a Gauss–Newton minimization is applied.^58^ Uniquely, a forward flux is defined as the average velocity magnitude over an initial time period and a backward flux for the remaining time interval.

Experiments included 10 sets of 3D head and neck squamous cell carcinoma patient DCE‐MRI data. While both the standard Tofts model $K^{\text{trans}}$ parameter and proposed forward flux follow similar trends, abrupt changes between neighboring slices and voxel are present in the $K^{\text{trans}}$ maps. Accounting for between voxel transport helped maintain the integrity of the forward flux estimations in the same regions.

Recently, Sinno et al.^48^ explored parameter recovery in tumor regions using a one‐compartment system with interstitial convection and diffusion with a global vascular input. This model is an extension of the standard Tofts model with additional diffusion and convection terms for contrast agent transport through the interstitium between neighboring voxels (Figure 2D). For vascular input, the model applies an AIF‐based approach. It, therefore, extends the approach proposed by Pellerin et al.^27^ with interstitial convection, or generalizes the model in Elkin et al.^47^ with a global AIF.

Sinno et al.^48^ reduce the complexity of the inverse problem by assuming radial symmetry, fitting 1D ROIs extending from the tumor center. Within each ROI diffusion is further assumed to be constant. The optimization approach is largely standard, applying a MATLAB ® nonlinear solver.

Experiments detail a set of 2D synthetic radially symmetric tumor models,^48^ and a set of 2D human cervical carcinoma xenograft DCE‐MRI data.^49^ Their results highlighted a successful differentiation of increased tumor periphery velocities along with considerable diffusivity at the tumor core. Due to assumed symmetry, tracer flow is restricted to along the radial direction only. Within the xenograft study $v_{e}$ is assumed constant, in contrast to their synthetic study where it was a free parameter. A sensitivity analysis showed no evidence of an impact on transport parameter fits for a fixed $v_{e}$ between 0.5 and 1. However, for smaller fixed $v_{e}$ the results became significantly different. As such, the validity of fixing $v_{e}$ is highly dependent on the influence it exerts on its co‐variant parameters.

### 4.3 Vascular convection

Several studies from a group at Cornell have developed a one‐compartment model with vascular convection.^50^, ^52^ The model introduces a spatially variable velocity coefficient, $u^{p}$, which acts to transport contrast agent through the vascular space between adjacent voxels (Figure 2E). Any diffusive transport between voxels is neglected due to the large magnitude of blood velocity.

For the inverse problem, this group^50^, ^52^ uses least‐squares optimization on the concentrations to fit for $u^{p}$ in each voxel. Specifically, an alternating direction method of multipliers with a conjugate gradient algorithm is applied. A regularization term based on the velocity gradient is employed, acting to enforce smoothness in the recovered velocity field.

Experiments comprise 3D synthetic datasets and 3D clinical data covering varied physiologies such as liver^52^ and kidney.^50^ For synthetic data production, a 1D nonlinear network of cylindrical models—solved using Poiseuille's law—are employed to represent the 3D microvascular network. To compare against the ground truth the convection and Navier–Stokes velocities are assumed to be equivalent.

For the synthetic data sets in Zhou et al.^50^ the introduced method achieved a smaller $u^{p}$ error than the Kety's method blood flow when compared with the ground truth $u^{p}$ values. While these approaches are clearly limited by an assumption of convection‐only transport, for intravascular indicators or highly vascularized well‐mixed systems this may well be justified.

### 4.4 Vascular convection and diffusion

The Cornell group, applying a similar inverse approach,^50^, ^52^ developed their method to include diffusive transport.^51^ Experiments include 3D clinical breast DCE‐MRI data, where Zhang et al.^51^ reported a more statistically significant distinction between malignant and benign breast tumors in $u^{p}$ than $K^{trans}$ from the Tofts model.

Sourbron^53^ introduced a one‐compartment model with both vascular convection and diffusion (Figure 2F). The inclusion of vascular diffusion increases the number of free parameters per voxel compared to convection alone, but actually simplifies the numerical problem by allowing for bi‐directional exchange at every voxel interface.

With this generalization, the inverse problem becomes linear and can be solved with standard matrix inversion methods. The unknowns of the discrete inverse system are rate constants at each voxel surface, which represent a combination of the diffusive and convective transport parameters. After solving the linear system for these rate constants, the results can then be converted back to convection and diffusion fields.

Experiments include a 2D synthetic data test case with a population AIF at selected boundary voxels, and the transport equations are solved by forward propagation of the linear system. Results showed that while the concentrations were reconstructed accurately from the data, the fitted parameter maps showed a deviation from the ground truth. These results indicate that the inverse problem in spatiotemporal modeling of DCE‐MRI is not in general well‐posed and multiple possible solutions exist that are compatible with the data. Strategies to resolving the degeneracy include refining the experimental conditions (e.g. faster injections or sampling), and/or adding regularizing constraints to select solutions with particular properties.

Within the DSC community, Liu et al.^54^ also propose a one‐compartment model with both vascular convection and diffusion (Figure 2F). The model introduces spatially variable velocity and diffusion coefficients, $u^{p}$ and $D^{p}$, respectively, which act to transport contrast agent through the vascular space between adjacent voxels.

To reduce the complexity of the estimation Liu et al.^54^ assume $u^{p}$ is incompressible. In consequence, this effectively constrains the system to have a constant volume fraction (Equation 3). For the inverse problem, Liu et al.^54^ use a stochastic gradient descent method to minimize the mean square error between the model and measurement concentration. Regularization terms based on gradients in diffusion and velocity are employed to enforce smoothness in recovered transport parameter fields. Their approach returns 3D maps of $u^{p}$ and $D^{p}$.

Experiments consist of two synthetic and 43 human stroke lesion DSC‐MRI data sets, both in 3D. Their synthetic datasets comprise of (1) purely convective and (2) purely diffusive transport, with various noise levels. The ground truth maps used for $u^{p}$ are derived using the inverse technique on a brain DSC‐MRI dataset, while $D^{p}$ are derived from apparent diffusion coefficient values.

Their synthetic investigations report a low error recovery of $u^{p}$ and $D^{p}$ that is robust to noise level increase. Within the stroke lesion study, Liu et al.^54^ consistently report lower velocity and diffusion values within lesion regions than normal regions. Additionally, their feature maps report a similar or improved interpretation of the stoke lesions when compared to standard perfusion maps.

The same group utilize partially supervised convolutional neural networks fitting the same system type (Figure 2F) to decrease computational time.^55^, ^56^ These methods^55^, ^56^ also apply velocity incompressibility and parameter regularization as in the original study.^54^ Across 10 of the same ischemic stroke data sets the new convolutional neural network‐based methodology showed greater distinction between lesion and normal regions than their previous work^54^ or standard perfusion metrics.

## 5 MULTI‐COMPARTMENTAL MODELS

While one‐compartment models have some practical utility, it is well‐known that most tissues require at least two compartments for an accurate description of their indicator concentrations. For instance, the assumption that intra‐ and extravascular spaces are well‐mixed, is in general not justified. Unfortunately, moving from one‐compartment to multi‐compartment spatiotemporal models comes with a step change in computational complexity.

In a multi‐compartment setting, the concentrations in the individual compartments are hidden and only the total concentration is directly accessible to measurement (Equation 2). Hence the multi‐compartment spatiotemporal model inherently requires solving a nonlinear system with hidden variables, or a linear system of higher order.

The literature is extremely sparse. Most spatiotemporal equivalents of standard multi‐compartment models only exist as theoretical proposals, or still rely on a global temporal input function, which does not model between‐voxel transport in the vasculature.

### 5.1 Interstitial diffusion and vascular input

Sainz‐DeMena et al.^59^ report a two‐compartment system with interstitial diffusion and a vascular input. This model applies a diffusion coefficient, D, which acts on the total tissue concentration. The vascular component is a nonnegligible plasma space $v_{p}$ with spatial variation and supplied by a global AIF.

Sainz‐DeMena et al.^59^ reduce the computational complexity of the inverse problem by considering 2D systems only, and assuming D everywhere is a constant known parameter. Minimization was implemented using a Trust Region Reflective algorithm, which handles sparse matrices efficiently.

Experiments include the 2D circular synthetic tumor previously proposed^27^, ^45^ and a 2D heterogeneous synthetic tumor with various noise levels. The proposed diffusion term enabled the method to consistently outperform the extended Tofts model for parameter accuracy in noise‐free scenarios. For systems with low measurement noise, the method showed significantly reduced fitting accuracy, particularly for $v_{p}$, compared to the relative stability of the extended Tofts model.

### 5.2 Vascular convection with exchange

Nævdal et al.^57^ implemented a two‐compartment system defined by Sourbron,^19^ modeling blood flow in arterial‐ and venous compartments and mono‐directional transport from arteries to veins by perfusion (Figure 2H). Their implementation employs a Darcy flow approach to define the arterial and venous velocities, with the intravoxel exchange from artery to vein mediated by a porous capillary space. Darcy flow is commonly used in porous media to describe pressure‐driven fluid flow.^60^ To relate this model to biology, porosity and permeability are interpreted in terms of compartmental volume fractions and the transport between compartments.

Concerning the inversion problem, Nævdal et al.^57^ decrease the computational complexity by applying a priori knowledge of either the permeabilities or porosity values to reduce the number of free parameters. For optimization, an Ensemble Kalman filtering method is applied, a popular method for parameter estimation in geoscience.^61^

Experiments included two synthetic 2D systems. The proposed method was applied for two separate investigations, either using known porosity values, or known permeability values. While the accuracy of the recovered porosity and permeability values are encouraging, the special cases presented apply very specific assumptions that would be inaccessible from a clinical DCE‐MRI dataset.

### 5.3 Three‐compartment models

A theoretical three‐compartment system has been proposed^19^ characterized by separate arterial and venous compartments with convective transport and an interstitial compartment with both convective and diffusive transport. These compartments interact with a mono‐directional exchange from artery to vein or interstitium and interstitium to vein (Figure 2I), following the picture of microvascular exchange involving extravasation at arterial ends of capillaries and reabsorption at venous ends. All the previously presented lower complexity compartment models are special cases of this general description. To the best of the authors' knowledge, there currently exists no implementation of a three‐compartment system.

## 6 DISCUSSION

This review has presented nine nested compartmental approaches that currently exist within the community. Of these nine models, seven have existing numerical implementations to recover between‐voxel transport coefficients, covering systems from pure interstitial diffusion to dual vascular convection with exchange. The presented approaches differ in complexity and applied assumptions, but all seek to extract spatial information that is inaccessible to single voxel modeling.

While all approaches build in methods of transport between voxels, either by diffusion and/or convection, some still assume a global vascular input to supply each voxel.^27^, ^45^, ^48^, ^49^ While convenient, this in some sense bypasses the key challenge of modelling transport to a voxel via exchange with neighboring voxels. Most recent work therefore has focused on removing the assumption of a global source.^46^, ^51^, ^52^, ^53^ Looking forwards, the further development of methods that do not require a global input is critical to achieve realistic models of indicator propagation across larger distances.

A prevailing problem limiting progression within this topic, is the availability of software implementations from previous studies. To the best of the authors' knowledge, there are no freely available software implementations for any of the presented methods. Consequently, in order to apply or develop any of the previously implemented approaches, researchers are faced with the major challenge of replicating the synthetic data and inversion methodology. Such re‐implementation is a significant time investment and acts as a barrier to the future development of otherwise promising methodologies. Moving forwards, increased efforts to publish algorithm and software details via open‐source sharing platforms such as GitHub would be invaluable. Not only will open science enable fast external implementation of existing methods but it can also help boost citations and collaboration opportunities.^62^ In recent years, there has been increased focus on open science within the perfusion imaging community via the forming of the International Society for Magnetic Resonance in Medicine Open Science Initiative for Perfusion Imaging an initiative and activity of the International Society for Magnetic Resonance in Medicine Perfusion study group.^63^ Contributions from Open Science Initiative for Perfusion Imaging and related projects cover challenges, code libraries,^64^ standardized data formats,^65^ and recommended lexicon naming conventions for DCE, DSC, and ASL.^66^, ^67^ Application of these and other software development guidelines^68^, ^69^, ^70^ to new contributions within the field will help to accelerate the pace of progression.

Another major hurdle to the development of useful spatiotemporal tracer kinetics modeling is the runtime of newly developed methods. Moving from single voxel modeling where each voxel may be fit independently, to a scenario where all voxels must be concurrently fit requires increased computational power. Some of the newly developed techniques apply assumptions within their models to reduce the number of free parameters per voxels, to reduce the computational requirement. While a useful exercise, new developments should focus on a reduction of physically inaccessible assumptions. Presenting an overview of computational runtimes for the implementations discussed in this review is not feasible without replicating the studies: apart from two studies^27^, ^45^ reported runtimes are unavailable.

Spatiotemporal tracer kinetic analysis would benefit from a fully generalized method for parameter reconstruction of any specified compartment model from tissue concentration data. Going forward, research in the field should focus on the development of methods that tackle the multi‐compartment inversion problem from small in silico test systems up to four‐dimensional in vivo datasets. This development to increasingly complex systems will incur a heavy computational load. For example, a fully spatial model with just two compartments has up to five free parameters to fit per voxel—compartmental convection and diffusion coefficients and an inter‐compartment exchange term. Such a high number of target parameters alongside a large volume of data appears to lend itself to machine learning approaches, such as the convolutional neural network methods proposed.^55^, ^56^ A relatively new branch of the machine learning field is Physics Informed Neural Networks (PINNs),^20^, ^71^ which incorporate the underlying system physics within the loss function to avoid unphysical solutions. The most applicable advance from the PINNs field is a method developed to identify parameters of the Navier–Stokes equations from concentration‐time data.^21^ A promising future direction for this work would be the adjustment of the PINNs network architecture to handle compartmental structures and tracer kinetics equations. Such a network would need to output the compartmental concentrations alongside relevant transport coefficients. To construct the physics‐informed aspect of the network, governing equations would be specified (e.g. any system in Table 1) and used to form residual equations. Appropriate steps for nondimensionalization would be required, alongside suitable activation functions and weighting schemes.^72^, ^73^ Due to the PINNs layout, modification of the output fields and system dimensions should be relatively straightforward, thereby creating a general inversion framework.

On a fundamental theoretical level, the spatiotemporal field currently lacks some broader understanding of the uniqueness of solutions, and to what extent this is affected by experimental conditions. This has been identified as a problem in several studies,^48^, ^53^ and is pivotal to the future development of inversion methods. Especially in multi‐compartmental systems, proof of unique solutions would increase confidence in results where recovered parameters show good agreement with concentration data. The extent to which an AIF is recoverable from the available measurement data, or whether it needs to be separately measured, is of particular interest. Additionally, further investigation into the dependence of uniqueness on experimental design is needed to reliably define solvable systems and conditions. Similar work on system design in standard perfusion quantification demonstrates that solutions degenerate if the indicator is not injected rapidly, or if sampling is too slow or too limited in duration.^74^, ^75^ It is likely that similar limitations are valid for spatiotemporal models, but no data currently exists to guide experimental design.

## 7 CONCLUSIONS

Nine nested model architectures for vascular‐interstitial tissues have been identified, although two of those have only been described theoretically. The most complex model currently implemented is a spatiotemporal two‐compartment exchange model. While these developments show promise, there exist unmet needs for model assumptions that apply to real‐world problems and for robust computational approaches to the inverse problem.

## FUNDING INFORMATION

This work is funded by an Engineering and Physical Sciences Research Council CASE Studentship (Ref: 2282622).

## CONFLICT OF INTEREST STATEMENT

Eve S. Shalom is supported by a CASE studentship from EPSRC with Bayer AG as the industry partner (Project Reference: 2282622) under supervision of Steven P. Sourbron, Sven Van Loo, and Amirul Khan.

## Data Availability

No new data were created or analysed during this study. Data sharing is not applicable to this article.
